# A preliminary investigation of nutritional intake and supplement use in Australians with myalgic encephalomyelitis/chronic fatigue syndrome and the implications on health-related quality of life

**DOI:** 10.29219/fnr.v65.5730

**Published:** 2021-06-07

**Authors:** Breanna Weigel, Natalie Eaton-Fitch, Rachel Passmore, Hélène Cabanas, Donald Staines, Sonya Marshall-Gradisnik

**Affiliations:** 1 The National Centre for Neuroimmunology and Emerging Diseases, Menzies Health Institute Queensland, Griffith University, Gold Coast, Australia; 2 Consortium Health International for Myalgic Encephalomyelitis, Menzies Health Institute Queensland, Griffith University, Gold Coast, Australia; 3 School of Medical Science, Griffith University, Gold Coast, Australia

**Keywords:** myalgic encephalomyelitis (ME), chronic fatigue syndrome (CFS), nutrition, nutritional intake, supplements, health-related quality of life (HRQoL)

## Abstract

**Background:**

Myalgic encephalomyelitis/chronic fatigue syndrome (ME/CFS) is a complex, multisystem illness without a currently recognized pharmacological treatment. Dietary supplementation and modification have been posited as potential management strategies; however, their efficacy is controversial.

**Objective:**

This study aimed to assess the nutritional intake and supplement use of Australian ME/CFS patients and the perceived effect on health-related quality of life (HRQoL) for the first time in an Australian patient population.

**Design:**

Between February 2019 and January 2020, ME/CFS patients across Australia volunteered in this cross-sectional study in response to online advertisements. Eligible respondents were invited to complete three online self-administered questionnaires investigating their supplement use, nutritional intake, and HRQoL. The study participants’ supplement use and nutritional intake were summarized and compared with the population data returned from the Australian Health Survey (2011–2012). Multiple linear regression analysis was also performed to determine the effect of participants’ supplement use and nutrient intake on HRQoL.

**Results:**

Twenty-four eligible ME/CFS patients (54.2% meeting the International Consensus Criteria, 79.2% female, mean age = 43.4 ± 10.5 years) completed the online questionnaires. Supplement use was highly prevalent among the study sample (87.5%) and considerably more common when compared with population data (31.9%). Daily total fats and caffeine intakes were significantly higher among ME/CFS patients when compared with the Australian population (*P* = 0.009 and *P* = 0.033, respectively), whereas daily intakes of total carbohydrates and alcohol were significantly lower (both *P* < 0.001). No consistent trends between nutrition and supplement use with patients’ HRQoL could be identified.

**Conclusions:**

The daily diet and supplement use of ME/CFS patients appear to vary considerably from those of the general Australian population. Although the role of nutritional intake and supplement use on ME/CFS patients’ HRQoL remains unclear, dietary changes and the use of supplements appear to be of value to ME/CFS patients.

## Popular scientific summary

- Supplement use is highly prevalent among myalgic encephalomyelitis/chronic fatigue syndrome (ME/CFS) patients, with vitamins and minerals as the most commonly taken supplements.
- The daily nutritional intake of ME/CFS patients is significantly different from that of the general Australian population, particularly the daily intake of fats, carbohydrates, alcohol, and caffeine.
- The relationship of nutritional intake and supplement use with health-related quality of life in ME/CFS patients remains unclear.

As a complex chronic illness, myalgic encephalomyelitis (ME) [also termed chronic fatigue syndrome (CFS)] has a considerable impact on patients’ daily activities and quality of life (1–4). The pathomechanisms underlying ME/CFS currently remain unknown and controversial due to the illness’ multisystem nature and heterogeneous clinical presentation (1–3, 5). A diagnosis of ME/CFS is dependent on persistent fatigue that cannot be explained by exercise or another fatiguing clinical syndrome, a significant reduction in the ability to perform one’s daily activities, and post-exertional neuroimmune exhaustion (2, 3, 6, 7). Additional symptoms may include bodily pain, autonomic dysfunction, cognitive impairments and neurosensory manifestations, unrefreshing sleep, and flu-like symptoms (2, 3, 5–8). Clinical presentation ranges from mild with a considerable reduction in patients’ ability to perform daily activities to very severe with patients bed-ridden and unable to complete basic daily tasks (6).

The onset of ME/CFS may be gradual or sudden, and while the illness’ pathogenesis remains contentious, onset often follows a viral or bacterial infection, physical trauma, exposure to toxins (such as pesticides or mycotoxins), or periods of prolonged stress (9–11). Presently, 25 different case definitions exist for ME/CFS with varying stringency (12). The Fukuda Criteria, Canadian Consensus Criteria (CCC), and the International Consensus Criteria (ICC) are the most widely accepted case definitions in the ME/CFS research and clinical arenas (12, 13). The availability of numerous case definitions with variable sensitivity precipitates inconsistency in ME/CFS prevalence estimates (8, 14). Generating accurate prevalence estimates is further complicated by the lack of validated and standardized disease biomarkers and laboratory tests to confirm a diagnosis of ME/CFS (2–4). Hence, ME/CFS prevalence estimates range from 0.01% to 7.62% (12).

Health-related quality of life (HRQoL) is significantly compromised in ME/CFS patients when compared with healthy individuals (4, 15–17). A large Australian study conducted at the National Centre for Neuroimmunology and Emerging Diseases (NCNED), which surveyed 480 ME/CFS patients meeting the Fukuda criteria from 2014 to 2018, observed significantly lower scores among ME/CFS patients across all HRQoL domains of the 36-Item Short-Form Health Survey (SF-36) when compared with population data collected from the Australian National Health Survey (4). Falk Hvidberg et al. similarly identified significantly lower HRQoL scores among 105 Danish participants with self-reported ME/CFS when compared with the general population (15). This investigation also compared the ME/CFS patients’ HRQoL scores with those of other chronic illness populations across Denmark, in which the mean HRQoL score for ME/CFS patients fell below that of 20 other chronic conditions, including rheumatoid arthritis, chronic renal failure, and several cancers (15).

As there does not currently exist a cure or commercially available pharmacological treatment specific for ME/CFS, symptom management and health optimization are utilized to attempt to help attenuate HRQoL deterioration (18–20). Dietary modification and supplementation have been posited as potential management strategies for ME/CFS (3, 18, 21, 22). New food intolerances or sensitivities appear to be a component of ME/CFS presentation, particularly intolerances to gluten, lactose, sugar, and alcohol (18, 23, 24). ME/CFS symptomatology also has similarities with various vitamin and mineral deficiencies (25). Although a systematic review published in 2017 concluded that there was insufficient evidence to implicate nutritional imbalances in the development of ME/CFS (26), nutritional status and intake appear to be more strongly correlated with HRQoL and symptom presentation in other chronic disease populations. Dietary modification and supplementation have been tenuously demonstrated as beneficial in improving patients’ quality of life for chronic conditions such as fibromyalgia (27), rheumatoid arthritis (28), and multiple sclerosis (29).

Dietary modification largely refers to the exclusion of trigger foods and food chemicals (18, 30). Due to the involvement of food intolerances in ME/CFS clinical presentation, some clinical guidelines recommend that suspected trigger foods be avoided by patients to prevent symptom exacerbation (18, 31, 32). Dietary interventions for ME/CFS management may also involve the inclusion of foods with perceived beneficial properties, such as polyphenol-rich dark chocolate [may assist in alleviating fatigue (30)] and herbal teas [may be involved in the improvement of sleep disturbances associated with ME/CFS (18)]. Also, although nutritional imbalances may not be causative of ME/CFS, supplements such as B vitamins, vitamin C, coenzyme Q (CoQ10), N-acetylcysteine, and glutathione may serve to improve the bioavailability of antioxidant compounds and, therefore, reduce inflammatory symptoms (33).

Randomized-controlled trials (RCTs) examining the effects of dietary interventions and supplement use on ME/CFS patients have, thus far, yielded inconsistent results (21). Additionally, dietary advice is minimal and varies across clinical guidelines (18–20, 31, 32, 34). Nonetheless, dietary changes and the use of supplements appear to be of importance to ME/CFS patients, who anecdotally report these strategies as being beneficial (18, 32). Therefore, supplements continue to contribute to some patients’ economic burden in the absence of conclusive evidence to suggest their effectiveness.

There is a paucity of literature about the impact of nutrition and supplement use on HRQoL in ME/CFS patients in Australia. Additionally, the use of supplements and the daily nutritional intake of Australian ME/CFS patients when compared with the general population is not well-described. As dietary modification and supplementation have been proposed as management strategies, it is hypothesized that the use of supplements and the daily nutritional intake of ME/CFS patients and will differ from that of the general Australian population. This pilot study, therefore, endeavors to characterize patients’ use of supplements and compare with that of the Australian population to elucidate the prevalence and importance of dietary supplementation among Australian ME/CFS patients. Additionally, this investigation will examine Australian ME/CFS patients’ nutritional intake and determine if this is significantly different from that of the general population. Finally, for the first time in an Australian ME/CFS patient cohort, this research will determine if supplement use and daily nutritional intake are significantly associated with HRQoL. It is anticipated that the outcomes of this study will inform future studies on management strategies for ME/CFS and, ultimately, improve patients’ quality of life.

## Methods

### Study design and setting

This cross-sectional study collected patient-level data regarding nutritional intake, supplement use habits, and HRQoL of Australian ME/CFS patients from February 2019 to January 2020. ME/CFS patients were recruited into the study through voluntary response to online advertisements released by the NCNED. Patients who responded to the online advertisements were subsequently assessed for eligibility (Supplementary Table 1) before being invited to complete the online questionnaires. This study received approval from the Griffith University Human Research Ethics Committee (GU:2019/1005 & GU:2016/807) and the Gold Coast University Hospital Human Research Ethics Committee (HREC/2019/QGC/56469).

### Online questionnaires

To determine eligibility, participants who responded to the online advertisements completed a survey through the online application, LimeSurvey (LimeSurvey, Carsten, Schmitz, Hamburg, Germany) (35). This questionnaire, which was generated by the members of the NCNED research team, enquired into patients’ sociodemographic information and extensively assessed patients’ ME/CFS symptoms to allow for classification of patients according to the Fukuda (36), CCC (37), and ICC (6) case definitions. Eligible respondents were subsequently invited to complete a second online questionnaire, also administered through LimeSurvey, which collected data regarding regular supplement use and HRQoL. Following this, the study participants completed the online self-administered Dietary Questionnaire for Epidemiological Studies (DQES) – distributed by the Cancer Council Victoria, Australia – which estimated patients’ daily nutritional intake by assessing the food and beverage consumption over the 12 months prior to completing the questionnaire (38). Participants who had completed the online questionnaires were then anonymized with an alphanumeric code. Stringent exclusion criteria were employed to reduce the potential for confounding variables that may explain the trends observed in this study (Supplementary Table 1). Following the elimination of participants that met the study’s exclusion criteria, the anonymized data were then exported to SPSS v26 (39) for statistical analysis.

### Study variables

#### Sociodemographic characteristics

Study participants disclosed their sociodemographic information, including: 1) gender (male, female, or other); 2) age (years); 3) age of illness onset (years); 4) height (cm) and weight (kg); 5) place of residence (by Australian state or territory); 6) current employment status (unemployed, part-time, or full-time); and 7) highest level of education obtained (primary school, high school, professional training [not university], undergraduate, or postgraduate). Participants’ height and weight were used to calculate their body mass index (BMI) (kg/m^2^), in which the study participants were subsequently categorized as underweight (<18.5), normal weight (18.5–24.9), overweight (25.0–29.9), or obese (≥30.0) as per the World Health Organization’s BMI classification system (40).

#### Supplement use

Participants’ regular use of supplements was recorded as either yes or no. Those currently taking supplements were subsequently required to list each supplement that they were taking and were categorized depending on the number of supplements being taken (one to three, four to six, or seven to 10). The study participants’ supplement-use habits were then compared with those of the general population. Population data were obtained from the Australian Health Survey: Nutrition First Results – Foods and Nutrients (AHS) (41). Between 2011 and 2012, 12,153 Australians participated in the AHS. To compare the proportion of ME/CFS patients taking supplements to that of the Australian population, the supplements reported by the patients were grouped into the following categories derived from the AHS: 1) multi-vitamins or multi-minerals; 2) single vitamins or single minerals; 3) lipid supplements; 4) herbal or plant-based supplements; 5) other nutritive supplements; and 6) other non-nutritive supplements. Supplement use was then further subdivided into the number of each supplement type taken by the study participants on a three-point scale: none, one to three, or more than four.

#### Health-related quality of life

HRQoL was examined through the use of items derived from the SF-36 (42). The items derived from the SF-36 utilized Likert scales from 0 to 100 to assess study participants’ HRQoL across seven domains: 1) physical functioning; 2) the role of limitations due to physical health problems; 3) bodily pain; 4) social functioning; 5) general mental health; 6) the role of limitations due to emotional problems; and 7) general health perceptions. The scores returned from the questionnaire are directly proportional to the study participants’ HRQoL for the corresponding domain.

#### Nutritional intake

Following the completion of the LimeSurvey questionnaires, study participants were invited to complete the online DQES. Dietary intake was assessed through 80 items across five domains, including: 1) cereals, sweets, and snacks; 2) dairy, meat, and fish; 3) fruits; 4) vegetables; and 5) alcoholic beverages. The items included within the DQES that assessed dietary intake were categorized as either ordinal or nominal. Ordinal items assessed the quantity of a particular food or beverage that was consumed per day, week, or month, or per portion of the food or beverage in question. Nominal items investigated the types of a particular food group that were typically included within the participants’ diet. Such nominal items were composed of both single-selection questions (where the most frequently consumed food type was queried) and multi-selection questions that requested all applicable options to be chosen. Participants’ daily nutritional intake was subsequently calculated based on the data derived from Nutrient Tables 2010 (NUTTAB) (43) and the Australian Food, Supplement, and Nutrient Database 2007 (AUSNUT) (44). The study sample means were then compared with those of the Australian population for the nutritional intake variables that were common between the DQES and the AHS.

### Statistical analysis

The data generated from the three online questionnaires were analyzed using SPSS v26. Sociodemographic and supplement use data are presented as the number of participants (percentage of total participants) unless stated otherwise. ME/CFS classification data are provided as the most stringent criteria met and are similarly presented as the number of participants (percentage of total participants). Data returned by the AHS are also provided where the relevant statistics were available and are presented as the percentage of AHS participants.

Nutritional intake data are presented as the sample mean ± standard deviation, as well as the means generated from the AHS. The daily nutrient intake and daily major and sub-major food group intake variables for the ME/CFS study sample were assessed for normality with the Shapiro–Wilk test. Due to limitations of the Shapiro–Wilk test, visual methods of determining normality (including histograms and Q–Q plots), as well as skewness and kurtosis values, were analyzed as quality assurance. In the event of conflicting results between the various methods used to determine normality, both parametric and non-parametric analyses were conducted and any loss or gain of significance that occurred is reported in the Results section. Most of the daily nutrient intake variables (except the daily intakes of alpha-linolenic acid, long-chain omega 3 fatty acids, total sugars, alcohol, folic acid, total folates, vitamin B6, and caffeine) were normally distributed and, thus, were analyzed with one-sample *t*-tests. The *t*-scores and *P*-values (α < 0.05) generated from these tests were then tabulated. All of the daily major and sub-major food group intake variables were deemed not normally distributed and were (in addition to the daily nutrient intake variables that were similarly not normally distributed as identified above) analyzed with one-sample Kolmogorov–Smirnov tests. The *D*-statistics and *P*-values (α < 0.05) returned from these tests are provided.

To assess the relationship of HRQoL with nutritional intake and supplement use, multiple linear regression analysis was performed for the seven SF-36 domains. The models generated for the seven SF-36 domains were adjusted for ME/CFS classification, age, gender, BMI, education, and employment. Following this, all supplement and daily nutrient intake variables were included in the forward stepwise procedure to generate the final model for each HRQoL domain. For all variables included in the final model, the unstandardized B (95% confidence intervals), standard error of B (SE[B]), standardized *ß*, *t*-value, and *P*-value are provided, in addition to the *R* ^2^-value, adjusted *R* ^2^-value, and *P*-value, for the model.

## Results

### Sociodemographic characteristics

Twenty-four ME/CFS patients completed the two online self-administered questionnaires. Table 1 summarizes the sociodemographic data of the study sample. The ICC case definition was met by over half of the study sample (54.2%), 29.2% met the CCC but not the ICC, and 4.2% met only the Fukuda diagnostic criteria. The average age of the study participants was 43.4±10.5 years and the average age of onset was 30.0±10.8 years. The majority of the study sample was female (79.2%), of normal weight (54.2%), and unemployed (70.8%). Most of the study participants had completed an undergraduate degree as their highest level of education (58.3%). In terms of location, those residing in Victoria (25.0%), New South Wales (20.8%), and Queensland (20.8%) occupied most of the study sample.

**Table 1:** Frequency of sociodemographic characteristics

	*N* (%)
**Classification**	
Fukuda	4 (16.7)
Canadian Census Criteria	7 (29.2)
International Consensus Criteria	13 (54.2)
**Gender**	
Female	19 (79.2)
Male	5 (20.8)
Other	0 (0.0)
**Age [years, mean ± standard deviation]**	43.4 ± 10.5
**Age of onset [years, mean ± standard deviation]**	30.0 ± 10.8
**Body Mass Index (kg/m^2^)**	
Underweight (<18.5)	0 (0.0)
Normal weight (18.5–24.9)	13 (54.2)
Overweight (25.0–29.9)	10 (41.7)
Obese (≥30.0)	1 (4.2)
**Location**	
New South Wales	5 (20.8)
Victoria	6 (25.0)
Queensland	5 (20.8)
South Australia	0 (0.0)
Western Australia	4 (16.7)
Northern Territory	0 (0.0)
Australian Capital Territory	2 (8.3)
Tasmania	2 (8.3)
**Employment**	
Unemployed	17 (70.8)
Part time	6 (25.0)
Full time	1 (4.2)
**Education**	
Primary school	0 (0.0)
High school	2 (8.3)
Professional training	2 (8.3)
Undergraduate	14 (58.3)
Postgraduate	6 (25.0)

### Supplement use

The frequency of supplement use among the study participants is outlined in Table 2. Most of the study participants were regularly taking at least one supplement (87.5%). This is a considerably larger proportion than the general population (31.9%). Of the total sample of ME/CFS patients, 66.7% took between four and 10 supplements. For each supplement group and subgroup where data from the AHS were available, the proportion of individuals taking the supplement in question was higher among ME/CFS patients than the general population.

**Table 2:** Frequency of supplement use

	Myalgic encephalomyelitis/chronic fatigue syndrome (N = 24) N (%)	Australian Health Survey (N ≈ 12,153)* %
**Supplement use**		
Yes	21 (87.5)	31.9
One to three	5 (20.8)	
Four to six	8 (33.3)	
Seven to 10	8 (33.3)	
No	3 (12.5)	68.1
**Multi-vitamins or multi-minerals**		
Yes	17 (70.8)	10.1
One to three	15 (62.5)	
Four or more	2 (8.3)	
No	4 (16.7)	
**Single vitamins or single minerals**		
Yes	17 (70.8)	
One to three	15 (62.5)	
Four or more	2 (8.3)	
No	4 (16.7)	
**Calcium**		
Yes	2 (8.3)	4.1
No	19 (79.2)	
**Magnesium**		
Yes	7 (29.2)	1.0
No	14 (58.3)	
**Zinc**		
Yes	8 (33.3)	0.4
No	13 (54.2)	
**Vitamin C**		
Yes	2 (8.3)	3.9
No	19 (79.2)	
**Vitamin D**		
Yes	8 (33.3)	4.3
No	13 (54.2)	
**Lipid supplements**		
Yes	7 (29.2)	14.0
One to three	7 (29.2)	
Four or more	0 (0.0)	
No	14 (58.3)	
**Fish oil**		
Yes	6 (25.0)	11.5
No	15 (62.5)	
**Evening primrose oil**		
Yes	1 (4.2)	0.5
No	20 (83.3)	
**Herbal or plant-based supplements**		
Yes	7 (29.2)	4.2
One to three	7 (29.2)	
Four or more	0 (0.0)	
No	14 (58.3)	
**Other nutritive supplements**		
Yes	4 (16.7)	1.3
One to three	4 (16.7)	
Four or more	0 (0.0)	
No	17 (70.8)	
**Fiber supplements**		
Yes	1 (4.2)	0.7
No	20 (83.3)	
**Protein or amino acid supplements**		
Yes	3 (12.5)	0.6
No	18 (75.0)	
**Other non-nutritive supplements**		
Yes	13 (54.2)	5.3
One to three	13 (54.2)	
Four or more	0 (0.0)	
No	8 (33.3)	
**Probiotics**		
Yes	10 (41.7)	1.0
No	11 (45.8)	
**Coenzyme Q10**		
Yes	5 (20.8)	0.8
No	16 (66.7)	
**Other supplements**		
Yes	2 (8.3)	0.4
No	19 (79.2)	

* *N* is equal to the total number of participants aged over 2 years; however, percentages are of the total number of participants aged over 19 years.

Note: The *N* values of the supplement subcategories add to 21 (total study participants who were taking at least one supplement) rather than the total 24 study participants; however, the percentages provided are of the total study sample.

Vitamin and mineral supplements were the most frequently taken supplements, in which multi-vitamin and multi-mineral supplements and single-vitamin and single-mineral supplements were taken by equal proportions of the study sample (70.8%). Vitamins and minerals were also the only supplement group where study participants were taking four or more of such supplements. The most frequently taken single vitamins or single minerals among the ME/CFS patients were zinc (33.3%), vitamin D (33.3%), and magnesium (29.2%). Although vitamin D was the most commonly taken single-vitamin or single-mineral supplement among Australians (4.3%), the use of this supplement (as well as all other single vitamin and single mineral supplements) was considerably higher in the ME/CFS sample.

Interestingly, lipid supplements were the most frequently taken supplement among the Australians included in the AHS (14.0%), where multi-vitamins and multi-minerals were taken by only 10.1%, and all single-vitamin and single-mineral supplements were taken by less than 5%. Non-nutritive supplements (being supplements without nutritional value, such as probiotics, CoQ10, or melatonin) were taken by 54.2% of the study sample compared with only 5.3% of the Australian population. The most noteworthy non-nutritive supplements were probiotics, which were taken by 41.7% of study participants but only 1.0% of the general Australian population.

### Daily nutrient intake

The daily intakes of fats and carbohydrates appear to be significantly different among Australian ME/CFS patients when compared with the general population (Table 3). There was a notably increased daily intake of total fats among ME/CFS patients when compared with the national average (*P* = 0.009). Significantly higher daily intakes of monounsaturated fats (*P* < 0.001), polyunsaturated fats (*P* = 0.001), linolenic acid (*P* < 0.001), and long-chain omega-3 fatty acids (*P* < 0.001) were observed among the ME/CFS study sample when compared with the general population. An additional non-parametric Kolmogorov–Smirnov test was performed, whereby the daily intake of saturated fats gained significance and was found to be significantly lower in the study participants (*P* = 0.037). The daily consumption of total carbohydrates, total sugars (including sugars in all forms), and starch was significantly lower among ME/CFS patients when compared with the Australian population (*P* < 0.001, *P* = 0.003, and *P* < 0.001, respectively). Following additional non-parametric analysis, dietary fiber was also found to be significantly higher among the ME/CFS sample when compared with the general population (*P* = 0.032). Interestingly, no significant difference in the daily energy intake was observed between ME/CFS patients and the general population (*P* = 0.118).

**Table 3:** Daily nutrient intake

	Myalgic encephalomyelitis/chronic fatigue syndrome (N = 24) Mean ± standard deviation	Australian Health Survey (N ≈ 12,153)^†^ Mean ± standard deviation	*t*	*P*
**Energy (kJ/day)**	7,936.24 ± 2,221.42	8,671.70 ± 5,735.84	-1.62	0.118
**Macronutrients**				
Protein (g/day)	83.01 ± 26.92	91.00 ± 70.22	-1.45	0.159
Total fat (g/day)	92.51 ± 32.07	73.80 ± 65.09	2.86**	0.009
Saturated fats (g/day)^§^	26.20 ± 10.12	27.70 ± 27.48	-0.72	0.476
Monounsaturated fats (g/day)	42.29 ± 17.12	28.40 ± 28.18	3.97***	<0.001
Polyunsaturated fats (g/day)	17.20 ± 7.56	11.40 ± 12.57	3.76**	0.001
Linoleic acid (g/day)	15.16 ± 6.99	9.40 ± 10.36	4.04***	<0.001
Alpha-linolenic acid (g/day)^‡^	1.50 ± 1.07	1.40 ± 1.85	1.27	0.079
Long-chain omega 3 fatty acids^‡^ (mg/day)	295.39 ± 214.59	281.40 ± 1,240.87	2.02***	<0.001
Total carbohydrate (g/day)	160.28 ± 67.14	225.90 ± 174.32	-4.79***	<0.001
Total sugars (g/day)^‡^	81.82 ± 40.36	102.90 ± 113.44	1.82**	0.003
Starch (g/day)	75.18 ± 43.66	118.30 ± 117.37	-4.84***	<0.001
Dietary fiber (g/day)^§^	25.76 ± 11.12	22.90 ± 22.72	1.26	0.220
Alcohol (g/day)^‡^	2.44 ± 4.90	14.40 ± 46.04	2.32***	<0.001
**Vitamins**				
Vitamin A retinol equivalents (µg/day)	999.49 ± 393.86	851.80 ± 2,535.38	1.84	0.079
Natural folate (µg/day)	352.83 ± 114.25	287.80 ± 285.55	2.79**	0.010
Folic acid (µg/day)^‡^	86.42 ± 105.90	192.60 ± 297.25	1.91**	0.001
Total folates (µg/day)^‡^	439.32 ± 185.10	480.80 ± 424.03	1.191	0.117
Vitamin B3 (mg/day)	21.21 ± 7.07	23.90 ± 18.44	-1.86	0.076
Vitamin B6 (mg/day)^‡^	1.17 ± 0.52	1.50 ± 2.15	1.68**	0.007
Vitamin B12 (mg/day)	2.99 ± 1.90	4.50 ± 6.95	-3.89***	<0.001
Vitamin C (mg/day)	129.71 ± 62.97	102.30 ± 146.61	2.13*	0.044
Vitamin E (mg/day)	13.24 ± 5.49	10.50 ± 11.58	2.45*	0.022
**Minerals**				
Calcium (mg/day)	789.27 ± 260.33	804.60 ± 798.30	-0.29	0.776
Iodine (µg/day)	119.40 ± 48.22	172.30 ± 151.96	-5.37***	<0.001
Iron (mg/day)^§^	11.99 ± 4.22	11.10 ± 9.79	1.04	0.311
Magnesium (mg/day)	441.40 ± 152.53	338.70 ± 261.37	3.30**	0.003
Phosphorus (mg/day)	1,370.25 ± 350.19	1,466.90 ± 970.27	-1.35	0.189
Potassium (mg/day)	3,525.32 ± 1,014.99	2,912.50 ± 2,247.53	2.96**	0.007
Sodium (mg/day)	1,891.40 ± 592.91	2,430.50 ± 1,875.58	-4.45***	<0.001
Zinc (mg/day)^§^	10.09 ± 4.02	11.00 ± 9.70	-1.11	0.278
**Caffeine (mg/day)**^‡^	336.78 ± 309.70	159.60 ± 246.32	1.44*	0.033
**Cholesterol (mg/day)**	283.67 ± 163.23	300.50 ± 364.40	-0.51	0.618

* *P* < 0.05,

** *P* < 0.01,

*** *P* < 0.001.

† , *N* is equal to the total number of participants aged over 2 years; however, percentages are of the total number of participants aged over 19 years.

‡ , Non-normally distributed variable; therefore, *D*-statistic is provided instead of *t*-score.

§ , Results of parametric analysis are provided; however, significance is gained with non-parametric analysis.

The bold values are statistically significant.

Significant differences were also observed in the nutrient make-up of the foods consumed. Compared with the general population, ME/CFS patients’ daily intakes of natural folate, vitamin C, and vitamin E appeared to be significantly higher (*P* = 0.010, *P* = 0.044, and *P* = 0.022, respectively), whereas daily intakes of folic acid, vitamin B6, and vitamin B12 were notably lower (*P* = 0.001, *P* = 0.007, and *P* < 0.001, respectively). In terms of minerals, the daily intakes of magnesium and potassium were significantly elevated among ME/CFS patients compared with the Australian population (*P* = 0.003 and *P* = 0.007, respectively). whereas the daily intakes of iodine and sodium were considerably lower (both *P* < 0.001). Subsequent non-parametric analysis found that iron intake was significantly higher and zinc intake was significantly lower among the study participants compared to the national average (*P* = 0.025 and *P* = 0.031, respectively). Daily caffeine intake among ME/CFS patients was also significantly higher compared with the general population (*P* = 0.033), and the daily alcohol intake was significantly lower (*P* < 0.001).

### Daily major and sub-major food group intake

Table 4 summarizes the study participants’ daily major and sub-major food group intake, in which each food category displayed statistical significance. Water intake was significantly higher in ME/CFS patients when compared with the Australian population (*P* = 0.042). Increases were observed in ME/CFS patients’ daily intakes of herbal tea, gluten-free bread, pasta and noodles, cream, and yoghurt (all *P* < 0.001). Fruit and vegetable consumption also appeared to be significantly higher among the ME/CFS patients when compared with the Australian population, in which the daily intake of all fruit and vegetable subgroups returned a significance level of *P* < 0.001 except the daily intake of bananas (*P* = 0.001). Interestingly, the daily intakes of total sugars, sausages, and processed meats were all significantly lower among the study sample (all *P* < 0.001). However, increases in the intake of eggs and chicken were observed in the ME/CFS patients when compared with the Australian population (both *P* < 0.001). Additionally, ME/CFS patients appear to consume less of all types of alcohol on a daily basis than the general population. For all types of alcohol assessed in the study (including light beer, heavy beer, white wine, and red wine), ME/CFS patients had significantly lower daily intakes when compared with the Australian population (all *P* < 0.001).

**Table 4:** Daily major and sub-major food group intake (g/day)

	Myalgic encephalomyelitis/chronic fatigue syndrome (*N* = 24) Mean ± standard deviation	Australian Health Survey (*N* ≈ 12,153)^†^ Mean ± standard deviation	*D*	*P*
**Non-alcoholic beverages**				
Water	1,472.67 ± 696.11	1,071.70 ± 1,535.88	1.39*	0.042
Herbal tea	308.73 ± 352.57	13.40 ± 106.36	2.20***	<0.001
**Cereals and cereal products**				
Gluten-free bread	13.39 ± 36.75	0.40 ± 6.57	2.33***	<0.001
Pasta and noodles	30.46 ± 26.37	17.90 ± 140.10	2.20***	<0.001
Breakfast cereals	21.15 ± 28.58	20.50 ± 61.02	1.81**	0.003
Porridge	8.07 ± 17.93	18.80 ± 109.84	2.12***	<0.001
Sweet biscuits	3.23 ± 7.52	8.20 ± 31.64	2.11***	<0.001
**Fats and oils**				
Butter	1.27 ± 1.99	1.70 ± 9.18	2.09***	<0.001
**Fruits and fruit products**				
Berries	16.60 ± 25.18	4.50 ± 36.71	2.21***	<0.001
Oranges	19.70 ± 30.75	11.50 ± 87.48	2.19***	<0.001
Bananas	25.80 ± 34.02	18.80 ± 72.54	1.95**	0.001
Pineapples	2.77 ± 4.57	1.20 ± 23.15	2.35***	<0.001
**Meat, poultry, and animal products**				
Eggs	29.98 ± 28.41	7.70 ± 34.80	2.02***	<0.001
Chicken	37.40 ± 33.90	24.30 ± 107.15	2.01***	<0.001
Sausages	5.91 ± 11.06	10.20 ± 68.59	2.16***	<0.001
Processed meat	3.70 ± 4.93	11.20 ± 49.39	2.23***	<0.001
Bacon	3.69 ± 5.65	2.80 ± 20.37	2.18***	<0.001
**Milk and dairy products**				
Yoghurt	37.63 ± 38.09	23.80 ± 107.57	2.02***	<0.001
Cream and sour cream	2.74 ± 4.64	1.60 ± 19.23	2.29***	<0.001
Flavored milk	0.00 ± 0.00	27.50 ± 190.99	2.73***	<0.001
**Vegetable products**				
Carrots	14.57 ± 12.28	8.80 ± 51.42	2.15***	<0.001
Pumpkin	11.29 ± 11.16	4.00 ± 33.95	2.22***	<0.001
Squash and zucchini	8.33 ± 9.94	1.20 ± 16.27	2.31***	<0.001
Mushrooms	8.70 ± 13.82	1.40 ± 16.51	2.28***	<0.001
Sweet corn	6.61 ± 7.52	4.60 ± 36.51	2.20***	<0.001
**Snack foods**				
Corn chips	4.38 ± 10.59	0.70 ± 13.74	2.35***	<0.001
**Sugar products**				
Sugar	1.25 ± 3.38	5.10 ± 17.43	2.40***	<0.001
Jam and related spreads	5.50 ± 15.47	2.20 ± 15.76	2.18***	<0.001
Chocolate	10.42 ± 13.81	6.90 ± 31.95	2.03***	<0.001
Other confectionary	3.38 ± 11.16	2.80 ± 18.83	2.16***	<0.001
**Alcoholic beverages**				
Light beer	0.29 ± 0.84	28.00 ± 308.67	2.60***	<0.001
Heavy beer	5.46 ± 16.13	88.70 ± 576.92	2.50***	<0.001
Red wine	11.43 ± 24.33	25.80 ± 167.81	2.15***	<0.001
White wine	20.46 ± 51.41	26.20 ± 155.97	2.12***	<0.001

* *P* < 0.05,

** *P* < 0.01,

*** *P* < 0.001.

† , *N* is equal to the total number of participants aged over 2 years; however, percentages are of the total number of participants aged over 19 years.

The bold values are statistically significant.

### Health-related quality of life

The multivariate analysis results for every HRQoL domain are presented in Table 5 and Table 6, corresponding to the physical health (physical functioning, the role of limitations due to physical health problems, bodily pain, and general health perceptions) and mental health (social functioning, general mental health, and the role of limitations due to emotional problems) domains, respectively. Three of the seven SF-36 domains returned significant regression models, including 1) the role of limitations due to physical health problems (adjusted *R* ^2^ = 0.733; *P* < 0.001); 2) bodily pain (adjusted *R* ^2^ = 0.544; *P* = 0.004); and 3) general health perceptions (adjusted *R* ^2^ = 0.743; *P* < 0.001). Complete statistics [including the unstandardized B, 95% confidence intervals, and SE(B)] for each HRQoL domain can be found in Supplementary Tables 2 to 8.

**Table 5:** Multivariate analysis of physical health-related quality of life domains

	‘Physical functioning’			‘The role of limitations due to physical health problems’			‘Bodily pain’			‘General health perceptions’		

	*β*	*t*	*P*	*β*	*t*	*P*	*β*	*t*	*P*	*β*	*t*	*P*
**Sociodemographic data**												
Age (years)	0.062	0.271	0.789	0.221	1.676	0.114	-0.028	-0.172	0.866	0.296	2.258*	0.040
Gender	0.089	0.400	0.694	0.208	1.693	0.111	0.028	0.177	0.862	0.306	2.341*	0.035
Body Mass Index (kg/m^2^)	-0.555	-2.628*	0.017	0.061	0.483	0.636	0.019	0.122	0.905	-0.033	-0.262	0.797
Employment	0.113	0.527	0.604	0.067	0.568	0.578	0.478	3.032**	0.008	-0.121	-0.979	0.344
Education	-0.014	-0.064	0.950	0.269	1.979	0.066	0.645	3.290**	0.005	-0.059	-0.382	0.708
**Dietary supplementation**												
Total number of supplements used												
Multi-vitamins or multi-minerals												
Single vitamins or single minerals												
Calcium												
Magnesium												
Zinc												
Vitamin C										0.300	2.238*	0.042
Vitamin D												
Lipid supplements												
Fish oil												
Evening primrose oil				-0.385	-3.197**	0.006						
Herbal supplements				0.618	5.162***	<0.001						
Other nutritive supplements												
Fiber supplements												
Protein or amino acid supplements												
Other non-nutritive supplements												
Probiotics												
Coenzyme Q10												
Other												
**Daily nutrient intake**												
Energy (kJ/day)												
Total protein (g/day)												
Total fat (g/day)												
Saturated fats (g/day)							0.860	4.609***	<0.001			
Monounsaturated fats (g/day)												
Polyunsaturated fats (g/day)												
Linoleic acid (g/day)												
Alpha-linolenic acid (g/day)										-0.543	-4.016**	0.001
Long-chain omega 3 fatty acids (mg/day)							-0.431	-2.663*	0.017			
Total carbohydrate (g/day)												
Total sugars (g/day)												
Starch (g/day)												
Dietary fiber (g/day)												
Alcohol (g/day)												
Vitamin A retinol equivalents (µg/day)												
Natural folate (µg/day)												
Folic acid (µg/day)												
Total folates (µg/day)				0.710	5.546***	<0.001						
Vitamin B3 (mg/day)												
Vitamin B6 (mg/day)												
Vitamin B12 (mg/day)												
Vitamin C (mg/day)												
Vitamin E (mg/day)												
Calcium (mg/day)										0.897	3.601**	0.003
Iodine (µg/day)										-0.602	-2.355*	0.034
Iron (mg/day)												
Magnesium (mg/day)												
Phosphorus (mg/day)												
Potassium (mg/day)												
Sodium (mg/day)												
Zinc (mg/day)												
Caffeine (mg/day)												
Cholesterol (mg/day)												
*R* ^2^			0.306			0.826			0.683			0.844
**Adjusted *R*^2^**			0.113			0.733			0.544			0.743
*P*			0.215			<0.001***			0.004**			<0.001***

* *P* < 0.05,

** *P* < 0.01,

*** *P* < 0.001.

The bold values are statistically significant.

**Table 6:** Multivariate analysis of mental health-related quality of life domains

	‘Social functioning’			‘General mental health’			‘The role of limitations due to emotional problems’		

	*β*	*t*	*P*	*β*	*t*	*P*	*β*	*t*	*P*
**Sociodemographic data**									
Age (years)	0.452	2.127*	0.049	0.442	2.038	0.057	0.231	0.980	0.342
Gender	0.059	0.302	0.766	0.089	0.420	0.679	-0.360	-1.649	0.119
Body Mass Index (kg/m^2^)	0.067	0.328	0.747	0.065	0.324	0.750	-0.152	-0.723	0.480
Employment	-0.061	-0.323	0.751	0.089	0.428	0.674	0.140	0.739	0.470
Education	0.848	2.957**	0.009	0.221	1.054	0.307	0.131	0.541	0.596
**Dietary supplementation**									
Total number of supplements used									
Multi-vitamins or multi-minerals									
Single vitamins or single minerals									
Calcium									
Magnesium									
Zinc									
Vitamin C				-0.422	-2.149*	0.046			
Vitamin D									
Lipid supplements									
Fish oil									
Evening primrose oil									
Herbal supplements									
Other nutritive supplements									
Fiber supplements									
Protein or amino acid supplements									
Other non-nutritive supplements									
Probiotics									
Coenzyme Q10							-0.536	-2.563*	0.021
Other									
**Daily nutrient intake**									
Energy (kJ/day)									
Total protein (g/day)									
Total fat (g/day)									
Saturated fats (g/day)									
Monounsaturated fats (g/day)									
Polyunsaturated fats (g/day)									
Linoleic acid (g/day)									
Alpha-linolenic acid (g/day)									
Long-chain omega 3 fatty acids (mg/day)									
Total carbohydrate (g/day)									
Total sugars (g/day)									
Starch (g/day)									
Dietary fiber (g/day)									
Alcohol (g/day)									
Vitamin A retinol equivalents (µg/day)									
Natural folate (µg/day)									
Folic acid (µg/day)	0.581	2.293*	0.036				-0.618	-2.447*	0.026
Total folates (µg/day)									
Vitamin B3 (mg/day)									
Vitamin B6 (mg/day)									
Vitamin B12 (mg/day)									
Vitamin C (mg/day)									
Vitamin E (mg/day)									
Calcium (mg/day)	0.795	3.349**	0.004						
Iodine (µg/day)									
Iron (mg/day)									
Magnesium (mg/day)									
Phosphorus (mg/day)									
Potassium (mg/day)									
Sodium (mg/day)									
Zinc (mg/day)									
Caffeine (mg/day)									
Cholesterol (mg/day)									
*R* ^2^			0.526			0.406			0.522
**Adjusted** R**^2^**			0.318			0.196			0.313
*P*			0.059			0.133			0.062

* *P* < 0.05,

** *P* < 0.01,

***P < 0.001.

The bold values are statistically significant.

Sociodemographic variables were significant predictors for HRQoL in the ‘bodily pain’ and ‘general health perceptions’ models. Employment (B = 21.728 [95% CI: 6.537–36.918], *P* = 0.008) and education (B = 19.846 [95% CI: 7.060–32.632], *P* = 0.005) were significant predictors for ‘bodily pain’ where age (B = 0.371 [95% CI: 0.019–0.724], *P* = 0.040) and gender (B = 9.763 [95% CI: 0.816–18.710], *P* = 0.035) were significant predictors for ‘general health perceptions’. Moreover, BMI (B = −2.976 [95% CI: -5.354–-0.597], *P* = 0.017) was significantly associated with ‘physical functioning’, and age (B = 0.922 [95% CI: 0.003–1.840], *P* = 0.049) and education (B = 21.823 [95% CI: 6.179–37.466], *P* = 0.009) were significant predictors for ‘social functioning’; however, neither of these models were significant.

In terms of supplement use, ‘the role of limitations due to physical health problems’ was found to be positively associated with the use of herbal supplements (B = 16.075 [95% CI: 9.438–22.712], *P* < 0.001) but negatively associated with the use of evening primrose oil supplements (B = −22.817 [95% CI: -38.030–-7.603.], *P* = 0.006). Additionally, the use of vitamin C supplements was positively correlated with ‘general health perception scores’ (B = 0.063 [95% CI: 0.003–0.123], *P* = 0.042). Opposingly, vitamin C supplements were found to be negatively correlated with ‘general mental health’ scores (B = −15.388 [95% CI: -30.494–-0.282], *P* = 0.046); however, this model was not significant (adjusted *R* ^2^ = 0.196, *P* = 0.133). Similarly ‘the role of limitations due to emotional problems’ was negatively associated with the use of CoQ10 supplements (B = −40.124 [95% CI: -73.317–-6.931], *P* = 0.021), yet this model was also insignificant (adjusted *R* ^2^ = 0.313, *P* = 0.062).

Regarding nutritional intake, negative associations were observed between daily long-chain omega 3 fatty acids intake and ‘bodily pain’ scores (B = −0.051 [95% CI: -0.092–-0.010], *P* = 0.017), daily alpha-linolenic acid intake and ‘general health perceptions’ scores (B = -6.707 [95% CI: -10.289–-3.125], *P* = 0.001), and daily iodine intake and ‘general health perceptions’ scores (B = −0.165 [95% CI: -0.315–-0.015], *P* = 0.034). However, positive associations were found between daily total folate intake and ‘the role of limitations due to physical health problems’ scores (B = 0.046 [95% CI: 0.029–0.064], *P* < 0.001), daily saturated fat intake and ‘bodily pain’ scores (B = 2.178 [95% CI: 1.176–3.180], *P* < 0.001), and daily calcium intake and ‘general health perceptions’ scores (B = 0.046 [95% CI: 0.018 –0.073], *P* = 0.003). Increased daily calcium intake was also correlated with higher ‘social functioning’ scores (B = 0.066 [95% CI: 0.024–0.107], *P* = 0.004), yet the ‘social functioning’ model was not significant (adjusted *R* ^2^ = 0.318, *P* = 0.059). Daily folic acid intake was positively associated with ‘social functioning’ scores (B = 0.118 [95% CI: 0.009–0.227], *P* = 0.036) but negatively associated with ‘the role of limitations due to emotional problems’ (B = -0.181 [95% CI: -0.338–-0.024], *P* = 0.026); however, the ‘the role of limitations due to emotional problems’ model was also not significant (adjusted *R* ^2^ = 0.313, *P* = 0.062).

## Discussion

This pilot study served to describe the supplement use and nutritional intake of Australian ME/CFS patients and compare with that of the general Australian population using the results of the 2011–2012 AHS. Furthermore, as dietary modification and supplementation currently exist as potential management strategies for ME/CFS (21, 45), this study aimed to collect patient-level data for the purpose of investigating the effects of dietary supplementation and nutritional intake on Australian ME/CFS patients’ HRQoL. A noteworthy finding of this study is that dietary supplementation is highly prevalent among ME/CFS patients despite having little observable effect on patients’ HRQoL. It also appears that the daily diet of ME/CFS is considerably different from that of the general Australian population.

The sample of ME/CFS patients in this study reflected the sociodemographic distribution of patients observed in previous cross-sectional studies (7, 9, 46–50). The majority of the study participants were female, of normal weight, and unemployed, which is consistent with the current literature (7, 9, 46–50). In congruence with other studies, the mean age of the study participants fell between 40 and 50 years, where the mean age of onset was similarly within the 30 to 40 years range (7, 9, 46, 49). Unusually, over three-quarters of the study participants had achieved at least an undergraduate-level education. This proportion is considerably larger than that observed in other studies (4, 7, 46, 47), which may be due to selection bias as a result of the study design relying on voluntary response.

All the study participants met the Fukuda criteria. The ICC case definition was met by over half of the study population, and among those fulfilling the ICC definition, all participants met the CCC diagnostic criteria. Thus, the majority of the study sample met at least the CCC definition. Although the Fukuda case definition has lower specificity than the CCC and ICC definitions, this is the most frequently used diagnostic criteria in the research and clinical arenas (13, 51, 52). Thus, by including patients meeting the Fukuda case definition, this study ensured that all subsets of ME/CFS were considered.

This study reports a novel finding that supplement use is highly prevalent among Australian ME/CFS patients when compared with the general population. However, there exists limited information in the current literature concerning supplement use among ME/CFS patients to validate these findings. Jones et al. (50) reported that the use of supplements or vitamins was significantly more prevalent among ME/CFS patients when compared with non-fatigued participants. Inversely, a study conducted by Boneva et al. found that the use of supplements in ME/CFS patients was lower than that in healthy participants (53). In support of this study, Dykman et al. reported that supplements were used by 100% of the study participants, which consisted of both ME/CFS and fibromyalgia patients (54). Thus, it is difficult to compare the results of this study with the available literature; as there is a lack of recently published studies, the sample sizes of the available studies are relatively small, and their results are likely influenced by confounding sociodemographic factors. The variability of supplement use among ME/CFS patients in the current literature serves to highlight heterogeneous supplement studies with highly variable outcomes and subsequent inconsistencies in understanding their effects and recommending their use (21).

Meanwhile, multi-vitamin and multi-mineral supplements did not appear to have a statistically significant association with any of the HRQoL domains despite the high prevalence of their use among the study participants. However, this observation should be confirmed in a future investigation with a larger study sample before definitively deeming these supplements ineffective in the management of ME/CFS. A multi-vitamin–multi-mineral supplement appeared to significantly improve fatigue, sleep, and autonomic function in ME/CFS patients in a 2014 study (55); however, there are few similar studies to confirm these findings. A 2002 RCT investigating the efficacy of a multi-nutrient supplement on fatigue, physical activity, and quality of life among ME/CFS patients did not return statistically significant results (56). Thus, the efficacy of multi-vitamins and multi-minerals as an adjunct in the management of ME/CFS symptoms remains controversial.

The use of single-vitamin and single-mineral supplements was similarly highly prevalent among the study participants; however, there were no clear correlations between any of the single vitamins or single minerals with patients’ HRQoL. However, vitamin C was positively correlated with patients’ ‘general health perceptions’ scores. Vitamin C has been proposed as a supplement recommendation to manage ME/CFS due to commonalities in the clinical presentation of ME/CFS and vitamin C deficiency, such as fatigue, depression, and cognitive impairment (25, 45, 57). Additionally, lower resting plasma levels of reduced ascorbic acid were observed in ME/CFS patients meeting the Fukuda criteria with a history of severe infection prior to ME/CFS onset when compared with healthy controls (58). Thus, further investigations are needed to elucidate the correlation between vitamin C and HRQoL in this study.

Intervention studies examining the efficacy of single vitamins or single minerals in ME/CFS symptoms are sparse. A 2015 RCT using vitamin D3 supplements reported no significant improvement in fatigue among ME/CFS patients meeting the Fukuda and CCC case definitions (59). A 2010 systematic review identified that NADH and magnesium supplements were associated with the improvements of ME/CFS symptoms, yet emphasized the importance of additional research to confirm these findings (60). The current literature suggests that CoQ10 supplementation may be a more promising approach to ME/CFS management (61–63). By improving mitochondrial function, the use of CoQ10 supplements may be useful for the management of depressive symptoms, neurocognitive dysfunction, and sleep disturbances associated with ME/CFS (61–64). Despite this, less than one-quarter of this study’s participants took CoQ10 supplements, and there were no significant positive associations with any of the three HRQoL regression models. Meanwhile, a negative correlation was observed between CoQ10 supplementation and ‘the role of limitations due to emotional problems’.

Apart from vitamin and mineral supplements, the only supplements displaying significant associations with HRQoL were herbal and evening primrose oil supplements. Herbal supplements were taken by 29.2% of the study population and had a positive association with ‘the role of limitations due to physical health problems’. An RCT of antioxidant pollen and pistil extract reported improvements in ME/CFS symptoms (including total well-being, fatigue, and depression) among patients meeting the Fukuda criteria (65). Despite this, a 2010 systematic review determined that there was insufficient evidence to implicate herbal supplements as effective in the management of ME/CFS (60). Inversely, the use of evening primrose oil supplements was negatively associated with ‘the role of limitations due to physical health problems’. This negative association between evening primrose oil supplements and HRQoL should be interpreted with caution, as only one of the study participants was taking evening primrose oil supplements. In the available literature, the use of evening primrose oil to ameliorate ME/CFS symptoms is poorly described (66). However, a 2007 review proposed that evening primrose oil supplements as a management strategy for ME/CFS patients by improving the bioavailability of long-chain fatty acids and, thus, immune function (67).

A systematic review published in 2017 concluded that although significant results were reported by some studies, no supplement was consistently associated with a significant improvement in ME/CFS patients’ condition (21). This is reflected in the results of this study, in which no supplement consistently displayed a significant positive relationship with HRQoL across the seven SF-36 domains. Therefore, due to disparate results in the literature and the lack of a clear association between supplement use and HRQoL in this study, the role of supplements in the management of ME/CFS remains unclear.

Another novel finding of this investigation is that the daily diet of ME/CFS patients appears to differ considerably when compared with the Australian population. To the best of the authors’ knowledge, there does not currently exist a study, national or international, that investigates the daily nutrient and food intake of ME/CFS patients (21). A study conducted by Goedendorp et al. ranking ME/CFS patients’ intakes of fruits, vegetables, and fiber found that patients’ intake of these food groups was not adequate to be considered healthy (68). Moreover, the significant differences in diet observed between ME/CFS patients’ and the general population in this study may be due to dietary changes in an attempt to manage ME/CFS symptoms. However, as patients’ ability to purchase and prepare foods may be hindered by the debilitating nature of ME/CFS (34), future research should investigate if the consumption of foods that have a longer shelf life or are easier to prepare (such as pre-packaged or frozen foods) is more prevalent among ME/CFS compared with the general population. Further, nutritional differences observed between this study sample and the Australian population may be due to the high prevalence of food intolerances and, therefore, food avoidance in ME/CFS patients (69).

Noteworthy differences between the study participants and the Australian population lay within the daily intakes of carbohydrate and fat, whereby ME/CFS patients appear to have a significantly lower daily intake of carbohydrates and significantly higher daily intake of fat when compared with the general population. Due to the nature of the DQES and AHS questionnaires, the daily intakes of total carbohydrates and total sugars could not be further sub-categorized, thereby limiting detailed comparison between the ME/CFS study sample and the general population. Despite this, the trends observed in this study may be attributable to common dietary modifications that are recommended for ME/CFS patients, such as ketogenic diets (low-sugar, high-fat diets), which may assist in alleviating inflammatory symptoms associated with ME/CFS (23). Additionally, the decreased carbohydrate intake among ME/CFS patients when compared to the general population may also explain the significantly lower folic acid and iodine intake within the study sample, due to the fortification of Australian flour with both of these nutrients (70). Significant associations between the daily intake of saturated fats, long-chain omega 3 fatty acids, alpha-linolenic acid, vitamins, and minerals with HRQoL were also observed. However, as there is limited literature pertaining to the role of nutrient intake in ME/CFS patients’ HRQoL, further investigation is warranted to determine if these associations have clinical significance.

The significant increase in daily caffeine intake among ME/CFS patients when compared with the general population is also worth noting. This is likely attributable to attempts to alleviate fatigue symptoms (18). However, as ME/CFS patients often experience heightened sensitivity to drugs and food chemicals (71), caffeine would be expected to exacerbate ME/CFS symptoms, such as anxiety and cardiovascular manifestations (72). The decreased daily intake of alcohol among ME/CFS patients compared with the Australian population was anticipated. Alcohol intolerance appears to be a component of ME/CFS presentation for many patients, likely due to its role in fatigue and cognition (9). Therefore, the significantly lower daily alcohol intake observed in the study sample is likely due to avoidance for the purpose of preventing symptom exacerbation. Yet, neither caffeine nor alcohol was significantly associated with patients’ HRQoL.

In terms of daily consumption of major and sub-major food groups, the most notable significant differences included herbal tea, yoghurt, gluten-free bread, and sugar. Patients’ significantly greater consumption of herbal tea and yoghurt may be explained as herbal and probiotic approaches to alleviating ME/CFS symptoms. Unfortunately, due to the nature of the DQES, herbal teas could not be further subdivided to determine if any specific herbal teas were associated with patients’ HRQoL. The increase of gluten-free bread and decrease of sugar in the study participants’ diet may be attributable to the dietary intolerances, in which food intolerance is often a component of ME/CFS presentation (46, 71).

The higher daily intake of fruits and vegetables among study participants when compared with the Australian population should be interpreted with caution, as only a limited number of fruit and vegetable products could be compared due to differences between the DQES administered to the study participants and the AHS. As dietary modification was found to be a common practice among ME/CFS patients in a previous observational study (69), the increased fruit and vegetable consumption in this study may be explained by the study participants being more conscious of their dietary intake than the general population. The 2017–2018 National Health Survey identified that only 5.4% of Australians aged over 18 years met the daily recommended intake for fruits and vegetables (73). Thus, further investigations should determine if ME/CFS patients are meeting the daily recommended intake requirements when compared with the general population to support the findings of this study.

This study is not without limitations. While the daily nutrient and major and sub-major food group intake data obtained from the AHS are assumed to be normally distributed based on the central limit theorem (74), normality tests could not be performed to confirm this, as individual data points from the AHS were not available. The unavailability of individual data points from the AHS also prevented inferential statistics from being generated for the supplement use component of this study. As a result, the use of supplements within the ME/CFS study sample and the general population could be described and compared; however, it was not possible to draw definitive conclusions about significant differences between these two cohorts. Furthermore, the lack of access to the complete AHS dataset hindered controlling for age, gender, education, and employment between the study sample and the general population. Thus, there is potential that this study overlooked sociodemographic factors that may explain the differences in supplement use and nutritional intake between the ME/CFS patients and the Australian population.

In addition, it is assumed that the DQES is an appropriate tool to measure the study participants’ daily nutrient and major and sub-major food group intake. However, as this study is the first to employ the DQES in an ME/CFS patient population, there does not exist another study to which the findings of this study can be compared. Also, although this food frequency questionnaire has been validated in Australian populations (75, 76), the study participants consisted only of adult Australian residents born in Australia, Greece, or Italy. Thus, the DQES may have less suitability to ME/CFS patients from other cultural backgrounds.

Additionally, there is potential for selection bias due to the nature of this study, and the study’s generalizability is compromised as a result of the small sample size. Therefore, the trends identified in this article should be confirmed with a larger sample size of ME/CFS patients with matched healthy controls to more accurately compare the nutritional intake and supplement use of ME/CFS patients to healthy individuals. This investigation also highlights the importance of validating the existing supplement recommendations for ME/CFS management, particularly regarding vitamins and minerals. As there is insufficient evidence to justify supplements as an ME/CFS management strategy, large international RCTs should be conducted to improve current recommendations. This would also permit investigation of a dose–response relationship between HRQoL and the supplements that displayed significance in this study. Such research is imperative, as the identification of effective supplements to complement management protocols may help patients prioritize financial resources.

## Conclusion

The objective of this cross-sectional study was to assess the daily nutritional intake and use of supplements among Australian ME/CFS patients and the effect of nutrition and supplements on patients’ HRQoL. Supplement use appears to be considerably more prevalent among ME/CFS patients when compared with the Australian population. Therefore, it appears that supplement use is a common practice among Australian ME/CFS patients. Additionally, the daily intake of fats is higher and carbohydrates is lower among ME/CFS patients when compared with the general population. This likely reflects the common dietary modifications that have been implicated in the alleviation of ME/CFS symptoms, thereby highlighting the perceived value of diet in ME/CFS management. No clear conclusions regarding the effect of nutritional intake and supplement use on ME/CFS patients’ HRQoL could be deduced from the results of this study. It is recommended that further research be pursued in this area with a larger sample size to validate the findings of this investigation.
